# Actions to prevent and identify fetal alcohol spectrum disorders to be implemented in general practice: a consensus

**DOI:** 10.3389/fmed.2024.1278973

**Published:** 2024-02-05

**Authors:** Sébastien Leruste, Alice Pouilley-Bax, Bérénice Doray, Thierry Maillard, Frédérick Monin, Coralie Loubaresse, Catherine Marimoutou, Michel Spodenkiewicz

**Affiliations:** ^1^Université de La Réunion—UFR Santé, Saint-Pierre, France; ^2^INSERM CIC-EC 1410, CHU of Réunion Island, Saint-Pierre, France; ^3^Laboratoire EPI (Etudes pharmaco-immunologiques), UFR Santé, Université de La Réunion, CHU (Centre Hospitalier Universitaire) de La Réunion, Saint-Denis, France; ^4^Service de Génétique, CHU (Centre Hospitalier Universitaire) de La Réunion, Saint-Denis, France; ^5^Centre Ressources TSAF (Troubles du Spectre de l’Alcoolisation Foetale), Fondation Père Favron, CHU (Centre Hospitalier Universitaire) de La Réunion, Saint-Pierre, France; ^6^Centre de Référence Anomalies du Développement et Syndromes Malformatifs Sud-Ouest Occitanie Réunion, Site Constitutif de La Réunion, Saint-Denis, France; ^7^SAF Océan Indien (SAF-OI), Saint-Louis, France; ^8^Moods Team, INSERM UMR-1178, CESP, Le Kremlin-Bicêtre, France; ^9^Department of Psychiatry, McGill Group for Suicide Studies, Douglas Mental Health University Institute, McGill University, Montréal, QC, Canada

**Keywords:** general practice, fetal alcohol spectrum disorders, early identification, consensus, Reunion Island

## Abstract

**Introduction:**

Fetal alcohol exposure is the most common preventable cause of non-genetic intellectual disability. Fetal Alcohol Syndrome (FAS) is characterized by intellectual disability and distinctive facial features and affects 0.1% of live births, representing approximately 800 cases per year in France. Fetal Alcohol Spectrum Disorder (FASD) are 10 times more common than FAS, with an estimated 8,000 cases per year, and are associated with behavioral and social maladjustment in both children and adults, as well as various malformations. General practitioners play a key role in preventing and identifying FASD through their involvement in pregnancy and child monitoring.

**Methods:**

Qualitative study using the Delphi method. Items were developed from the literature and semi-structured interviews with field professionals and health institutions. A panel of multi-professional experts, mostly general practitioners, was recruited.

**Results:**

24 initial actions were submitted to the experts. At the end of the first round, six actions reached a consensus and six were reformulated for the second round. At the end of the second round, three actions reached a consensus, for a total of 11 consensus actions. Four of these actions seem particularly relevant for rapid implementation, namely systematic proposal of pre-conceptional consultations for women planning pregnancy, systematic identification of environmental factors during child monitoring, systematic distribution of information on fetal alcohol exposure during pre-conception or early pregnancy, and the publication of a leaflet for general practitioners on the identification of children with FAS or FASD and the contact details of relevant associations.

**Conclusion:**

Prevention and identification of FASD can be improved through short and general training supports for general practitioners. Early screening of FASD is crucial for children, and should be maintained throughout their monitoring. This study could be used for communication and dissemination of information based on the consensus obtained.

## 1 Introduction

Although the cause of FASD is known, prenatal fetal alcohol exposure represents the most common cause of non-genetic intellectual disability (1). In France, 12% of pregnant women declared in 2017 that they had consumed alcohol during their pregnancy, regardless of whether they knew they were pregnant (2).

Among disorders resulting from prenatal alcohol exposure, Fetal Alcohol Syndrome (FAS) is the most typical and least difficult to diagnose. It associates a characteristic facial dysmorphia, a delayed growth in height and weight and neurodevelopmental disorders of varying severity. It accounts for 0.1% of births, or around 800 cases a year.

The toxicity of alcohol during pregnancy can vary according to the quantity of alcohol consumed, the period and duration of exposure. The various physical and neurodevelopmental impairments caused by exposure to alcohol during pregnancy are heterogeneous and grouped together under the term Fetal Alcohol Spectrum Disorder (FASD). Four distinct diagnostic categories were established in 1996 by the Institute of Medicine (IOM): Fetal Alcohol Syndrome (FAS), partial Fetal, Alcohol Syndrome (pFAS), Alcohol-Related Neurodevelopmental Disorders (ARND), and Alcohol-Related Birth Defects (ARBD) (3). FASD is 10 times more common than FAS, accounting for 7.7‰ of births worldwide (4), with an extrapolation of 7,700 births cases per year in France.

These disorders manifest themselves from birth through adulthood (5). There is an increased risk of intellectual disability, psychiatric disorders, and secondary comorbidities or disabilities such as addictions, delinquency, and criminal behavior (6).

The meta-analysis by Popova et al. (4) identified over 400 comorbid conditions in people with Fetal Alcohol Spectrum Disorder. The most common are congenital malformations, chromosomal deformities and abnormalities, and mental and behavioral disorders.

In 2015, La Réunion was designated as a pilot region by the government to set up a program for the prevention and management of fetal alcohol spectrum disorders. An FAS prevention action plan was implemented between 2016 and 2018. It was articulated around six axes (Appendix 1) including professional training and the implementation of a care pathway for the women and children concerned (7).

General practitioners appear to be key players in the prevention and detection of FASD, through their involvement in pregnancy and child follow-up. On the other hand, studies suggest that GPs sometimes feel incompetent in screening and management (8, 9). In 2021, an action research project was launched. A review of the literature and exploratory research were carried out to explore the obstacles and motivations faced by GPs in preventing and identifying FASD. The doctors interviewed revealed a lack of theoretical and practical knowledge on the subject, as well as insufficient knowledge of the care pathway and structures for diagnosing and managing FASD (10). For the second stage of the action research, several actions were identified, but a consensus was needed to prioritize relevant actions in general practice.

## 2 Materials and methods

### 2.1 Type of study

The Delphi method was used, a systematic and interactive prospecting method based on a panel of experts. It enables a group of experts to be questioned on a defined subject in order to reach a consensus (11). Each expert is interviewed individually and anonymously on the basis of an initial questionnaire.

### 2.2 Population

The use of an expert panel should not be confused with the consultation of a group of high-ranking scientists. An expert is defined as a person with sufficient knowledge of the subject because of professional or personal involvement in the issue (11, 12).

For this study, the recruitment of experts implied knowledge in the field of FASD through their professional practice or personal involvement in this issue. The inclusion criteria for the various players who were sought to make up the expert group were:

- Outpatient general practitioners.
- Other healthcare professionals involved in the care of women of childbearing age and infants.
- Socio-educational actor working with children with Fetal Alcohol Spectrum Disorder.
- Families and patients with Fetal Alcohol Spectrum Disorder.

As far as general practitioners are concerned, the inclusion of those who have or have had a practice focused on child health and/or women’s health and/or addictology was favored.

Recruitment was network-based. Experts were invited individually by email (Appendix 2), explaining the objectives of the study and the Delphi method. They were integrated into the network after having familiarized themselves with the elements and agreed to the study’s modalities.

**Figure 1:**
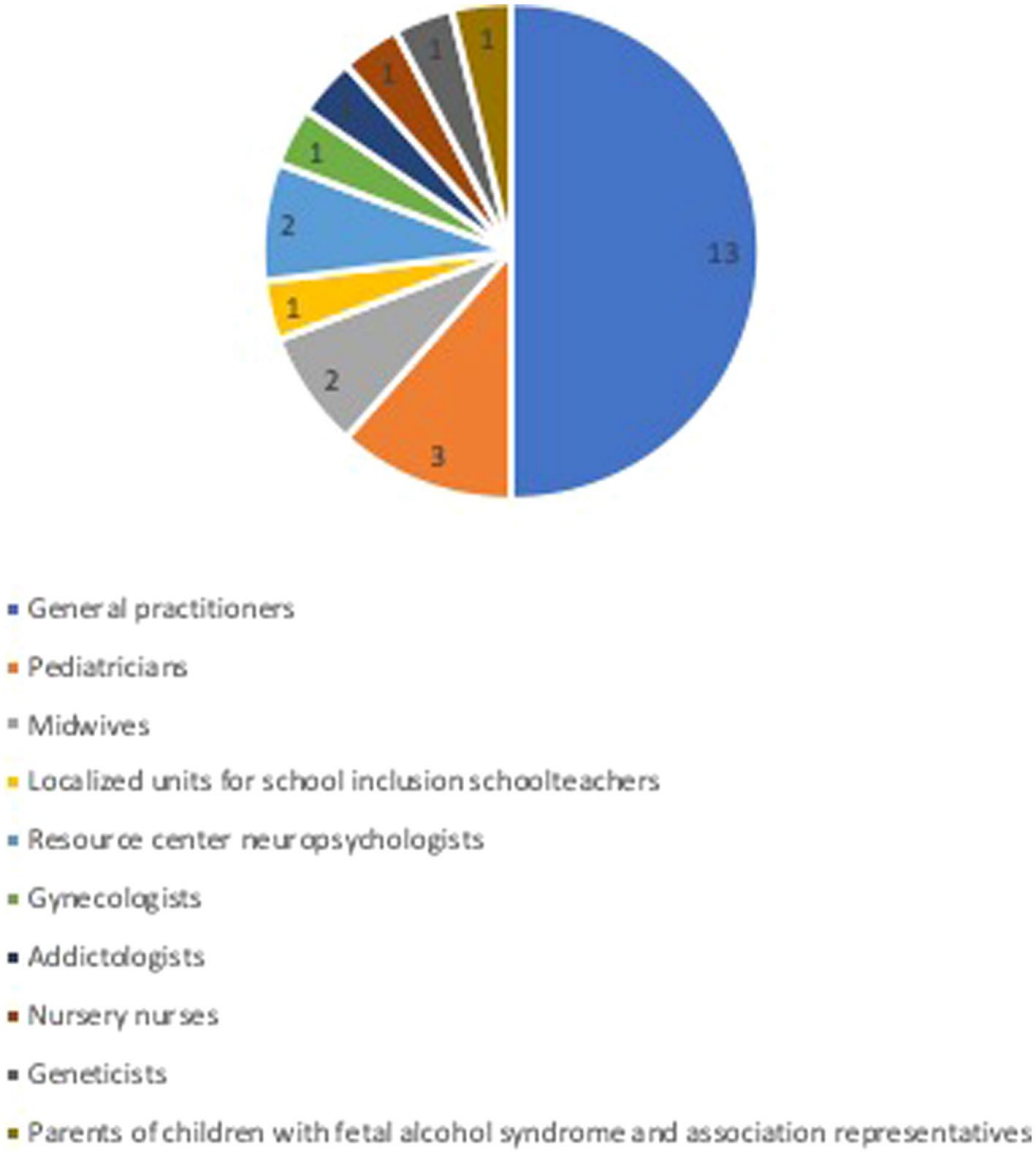
Expert panel.

The expert group is made up of 26 experts, including seven men and 19 women. They were mainly general practitioners (13), but also pediatricians (3), midwives (2), Localized units for school inclusion schoolteacher (1), resource center neuropsychologists (2), gynecologist (1), addictologist (1), nursery nurse (1), geneticist (1), and parent of children with fetal alcohol syndrome and association representatives (1) (Figure 1).

### 2.3 Data collection

#### 2.3.1 Drawing up the initial questionnaire

The choice of evidence-based prevention actions implemented by primary care teams was based on the literature review carried out prior to this work. This selection was supplemented by interviews with local FASD healthcare providers to identify non-evidence-based actions that have been implemented. What all these initiatives had in common was that they aimed to prevent and identify FASD in couples of childbearing ages, as well as in pregnant women and children. Twenty-six “initial” actions emerged from this work, and were proposed to the experts. Following the initial questionnaire, a number of actions could already be agreed upon.

The comments were analyzed using a qualitative research approach based on thematic analysis. This enabled us to identify various themes for the second questionnaire.

#### 2.3.2 Expert consultation

The application chosen for submitting the questionnaires was Google Forms®. Invitations to complete the questionnaires were sent by email, with a link to the questionnaire.

#### 2.3.3 First round

For each action, the experts were asked to indicate their degree of agreement on each action on a Likert scale ranging from 1 (complete disagreement) to 9 (complete agreement), and then to comment on each action to argue their opinion.

First, for each action, the median of responses was calculated, enabling the position to be judged in relation to the proposed action:

Median ≤ 3: disagreement.
Median between 4 and 6: equivocal.
Median ≥ 7: agreement

Secondly, the degree of convergence within the group was calculated in order to define the so-called consensual actions. Literature data suggest a rate of agreement between experts ranging from 51 to 80% (3). In our study, this rate of agreement was set at 70%. Thus, if 70% of expert responses were greater than or equal to the median, the action was said to be consensual. For actions with a rate of agreement below 70% of the median, the actions were said to be non-consensual. Comments on these actions were analyzed individually or in combination to compose the second questionnaire.

#### 2.3.4 Second and final round

The statistical analysis used to define the consensus actions was carried out in the same way as in the first round. The researchers (AP-B, SL, and MS) considered that the number of actions on which there was consensus in the initial questionnaire and the second questionnaire was sufficient for consensus building.

The aim of analyzing the comments from the second questionnaire was to open up avenues of reflection for the implementation of consensual actions or other research projects. Comments were analyzed by theme for each action individually.

### 2.4 Ethical and regulatory criteria

A request for compliance with the MR004 reference method was made to the Commission Nationale de l’Informatique et des Libertés (CNIL) prior to the study, and registered under n°2,226,567.

The data were collected anonymously via a data entry mask edited with the Google Forms® application.

## 3 Results

### 3.1 First round results

**Figure 2:**
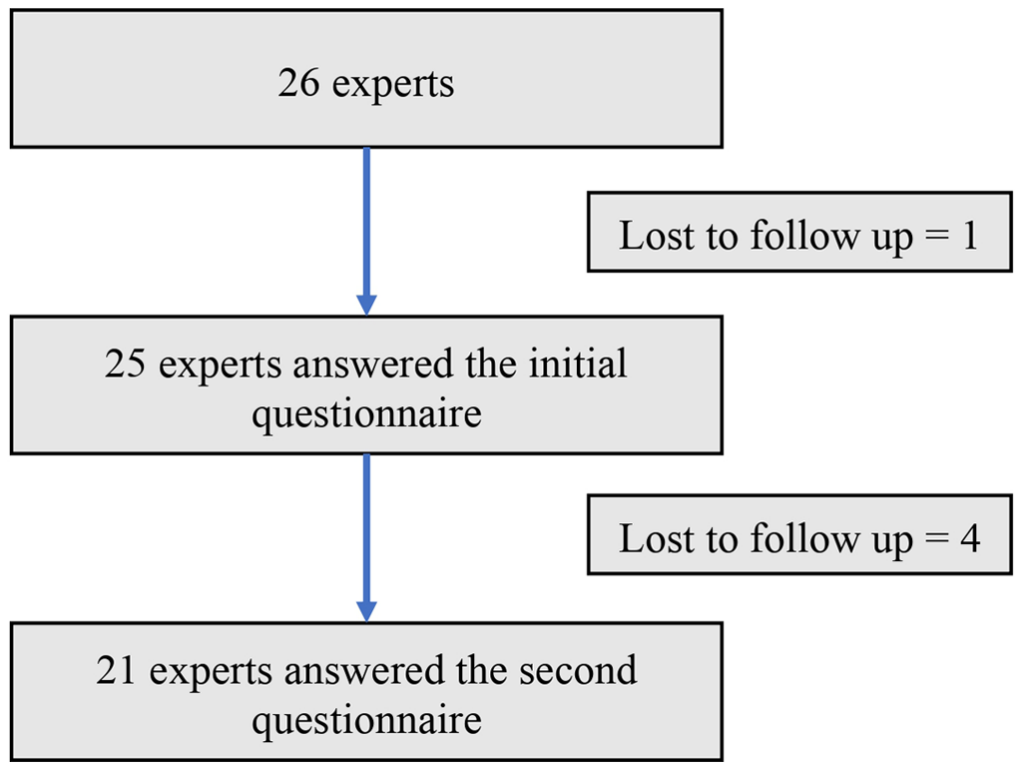
Flow chart.

Twenty-six invitations to reply to the first questionnaire were sent to the experts on July 11 2022. Experts were given 3 weeks to respond. A reminder was sent on August 1, 2022 to experts who had not responded to the initial questionnaire. A 2-week extension was granted, as several experts were on vacation during this period and did not receive their mail. Twenty-five experts were able to respond to the first questionnaire between July 11, 2022 and August 22, 2022 (Figure 2).

Among the 24 actions proposed in the first round:

- 22 actions were approved by experts, eight of them by consensus.
- Two actions were deemed equivocal by the expert group.
- There was no disagreement among the group.

Here are the eight consensus actions from the first round:

- Systematically offer a pre-conception consultation to young women wishing to become pregnant.
- Set up multidisciplinary structures with medical, psychological and social players.
- Integrate FASD into general practitioner training courses on neurodevelopmental disorders (NDD).
- Systematically identify environmental factors during the follow-up.
- Produce an information leaflet for general practitioners on identifying children with FAS or FASD, with contact details for associations.
- Enable GPs to easily refer pregnant women with alcohol addiction to competent weaning structures, by creating priority access for pregnant women to these structures.
- Systematic distribution of an information leaflet on fetal alcohol syndrome when folic acid is prescribed pre-conceptionally or in early pregnancy.
- Create Continuing Professional Development (CPD) training courses on FASD for general practitioners.

Sixteen actions did not achieve consensus in the first round. Three actions were dropped after analysis of the first questionnaire, as analysis of the comments did not reveal any new proposals. Three other actions were dropped because their objectives were close to those of the consensus actions. These were to discuss the best time to distribute an information document on fetal alcohol syndrome, and which training medium should be used to train GPs. Three actions were reformulated individually in the second questionnaire. Lastly, the comments on seven actions were analyzed by theme, leading to the proposal of three new actions to complete the second questionnaire (Table 1).

**Table 1:** Summary of first-round results.

Proposed actions—TOUR 1	M	DC	R
Conduct brief interventions on alcohol consumption among women of childbearing age, using standardized questionnaires (Example: DETA, AUDIT).	8	52%	AR
Systematic distribution of an information leaflet on fetal alcohol syndrome by general practitioners during consultations with women of childbearing age.	7	52%	DA
The doctor monitoring the pregnancy should receive feedback on the 4th-month prenatal interview with the midwife (example: letter).	9	68%	AR
Systematically offer a preconception consultation to young women wishing to become pregnant	9	72%	CA
Set up multidisciplinary structures with medical, psychological and social professionals dedicated to monitoring pregnant women who drink alcohol.	9	72%	CA
Develop a local smartphone application for the identification of Fetal Alcohol Syndrome (FAS) and Fetal Alcohol Spectrum Disorder (FASD). This application would help refer patients according to a probabilistic score based on dysmorphia and child development.	9	62.6%	AR
Integrate FASD into general practitioner training courses on neurodevelopmental disorders (NDD).	9	88%	CA
Systematically identify environmental factors during the child’s follow-up.	9	84%	CA
Ask about alcohol consumption several times during the pregnancy follow-up, using open-ended questions (e.g., when giving advice on diet, when filling in the maternity booklet, etc.).	9	60%	AR
Produce an information leaflet for general practitioners on identifying children with FAS or FASD, with contact details for associations.	9	80%	CA
Implementation of a screening program for FASD during child follow-up in general practice.	8	56%	AR
Posters on fetal alcohol syndrome in all general practices.	9	62,5%	DA
Enable GPs to easily refer pregnant women with alcohol addiction to competent weaning structures, by creating priority access for women.	9	88%	CA
Systematic distribution of an information document on fetal alcohol syndrome when a biology test involving the detection of pregnancy hormone (HCG) is prescribed.	8	56%	DA
Setting up a university diploma (DU) for general practitioners on NDT, part of which would focus on fetal alcohol spectrum disorders.	9	56%	DA
Routine screening for neurodevelopmental disorders in general practice for all children at age 3.	9	60%	AR
Identification of women at risk of exposure to alcohol during a future pregnancy by the general practitioner. These patients are then offered contraception, with regular assessment of compliance and tolerance.	5	70.8%	DA
Screen ALL pregnant women using standardized questionnaires in the waiting room (e.g., T-ACE, AUDIT-C).	7	60%	AR
Create Continuing Professional Development (CPD) training courses on FASD for general practitioners.	9	72%	CA
Enhance the value of addictology consultations in general practice by introducing a specific rating system.	8	66,7%	DA
During information and awareness campaigns on fetal alcohol syndrome, emphasize the seriousness of the clinical picture and the consequences for families and caregivers, in order to have a greater impact.	7	62.5%	AR
Systematically measure drug consumption at every pregnancy check-up via the maternity record (and not just at the start of pregnancy).	8	64%	AR
Systematic distribution of an information leaflet on fetal alcohol syndrome when folic acid is prescribed pre-conceptionally or in early pregnancy.	8	80%	CA
Identify high-risk drinking situations during pregnancy follow-up, using standardized self-questionnaires during general medical consultations (e.g., T-ACE, AUDIT-C).	7	58.4%	AR

M, Median. DC, Degree of convergence or percentage of responses within the median interval. R, Result; CA, Consensus action; DA, Discontinued action; AR, Action reworked for second round.

#### 3.1.1 Comments analysis

A thematic analysis of the comments revealed a number of themes for reflection: prevention by the GP, the implementation of multidisciplinary follow-up, child follow-up, information documents, GP training, and the use of standardized questionnaires during consultations.

Here is a summary of this analysis:

Prevention by general practitioners must be targeted. It should not be based solely on “childbearing age,” but should identify women with a desire to become pregnant. It was also important to make the partner aware of fetal alcohol syndrome. The partner’s role is to accompany and support the woman during pregnancy. It would be interesting to look for alcohol consumption in the partner, and to quantify it.
During pregnancy monitoring, the prevention and detection of FASD must be a multidisciplinary team effort (doctors, midwives, addictologists, social workers, etc.). The information transmitted between the various professionals must be relevant to the patient’s interests, and in agreement with her.
Screening for FASD should be carried out at a very early stage, from the child’s first months of life, well before starting school, and then during the child’s follow-up. The health record seems to be the ideal medium for screening for neurodevelopmental disorders, including FASD. For example, the health record could be modified to include warning signs during compulsory visits.
The systematic distribution of an information document on fetal alcohol syndrome was approved by the experts, but they agreed on the importance of distributing it at the right time. The availability of this information to patients during HCG prescription or urine purchase of a pregnancy test was discussed by the experts. On the other hand, the pre-conception consultation, the prescription of folic acid, or when a couple expresses a desire for pregnancy, seem to be key moments for distributing the document.

As far as training general practitioners in FASD is concerned, there is a need for “broad” training. According to the experts, the introduction of a university diploma (DU) specific to FASD could encourage the training of doctors only interested and involved in the subject, and thus a minority of healthcare professionals.

The idea would be to use shorter training materials such as Continuing Professional Development (CPD) and to deepen their knowledge of FASD in more general training courses, such as those on child follow-up or neurodevelopmental disorders.

The use of standardized questionnaires to screen for alcohol consumption has been validated by the experts. On the other hand, it should provide an opportunity for discussion and support with the general practitioner. The use of simplified questionnaires during consultations was proposed.

### 3.2 Second round results

Twenty-five invitations to reply to the second questionnaire were sent to the experts on October 10, 2022. Experts were given 3 weeks to respond. Two reminders to complete the questionnaire were sent out during this period. Twenty-one experts were able to respond to the second questionnaire between October 10, 2022 and October 31, 2022. Four experts did not reply. The reasons for their non-participation have not been identified (Figure 1).

Of the six actions proposed in the second round, all six were approved by experts, including three with consensus:

- Use the child’s health record as a tool for early detection of neurodevelopmental disorders, including fetal alcohol spectrum disorders.
- During pregnancy monitoring, look for the use of various toxic substances on several occasions, not just during the consultation.
- Set up transmission of the minutes (by post or email via secure messaging system) of the 4th-month prenatal interview, with the patient’s consent.

The total number of actions (eight actions in the first round and three actions in the second round, i.e., 11 actions) was deemed sufficient to build consensus, enabling the Delphi Round to be closed.

Unlike the initial questionnaire analysis, comments on each action were analyzed individually (Table 2).

**Table 2:** Summary of second-round results.

Proposed actions—TOUR 1	M	DC	R
Use the child’s health record as a tool for early detection of neurodevelopmental disorders, including Fetal Alcohol Spectrum Disorder (FASD), for example, by including the search for “warning signs” in the child’s compulsory follow-up visits.	9	81%	CA
Creation of an application or website for general practitioners to help them screen for and refer neurodevelopmental disorders, including alcohol spectrum disorders.	9	52%	DA
Research and quantify the partner’s alcohol consumption when a couple is planning a pregnancy, because men are also involved in preventing fetal alcohol syndrome.	9	67%	DA
During pregnancy follow-up, look for the consumption of various toxic substances on several occasions, not just during the initial consultation, and record it in the patient’s medical record.	9	71%	CA
During general medical consultations, conduct brief interventions on alcohol consumption among women and couples with a desire to become pregnant, using standardized questionnaires (Example: DETA, AUDIT).	8	52%	DA
Implement transmission of the minutes (by post or email via secure messaging system) of the 4th-month prenatal interview with the midwife to the doctor monitoring the pregnancy, with the patient’s consent.	9	86%	CA

M, Median. DC, Degree of convergence or percentage of responses within the median interval. R, Result; CA, Consensus action; DA, Discontinued action.

#### 3.2.1 Comments analysis

With regard to the use of the health record as a tool for the early detection of neurodevelopmental disorders, including FASD, the expert group reached a consensus. On the other hand, it was emphasized that doctors should be trained to identify these disorders. The choice of medium (health record) was discussed by five experts, as the group felt it was important to be vigilant about respecting medical confidentiality with regard to fetal alcohol. The use of computerized medical records was suggested by one expert.

There was no consensus on the creation of an application or website for general practitioners to help them screen for and refer neurodevelopmental disorders, including FASD. The complexity of the tool was underlined by the group. The use of a website for identification during consultations was judged by several experts to be time-consuming. If a website were to be developed, it would have to be quick and easy to use. The “Antibioclic®” site, which concerns the rational prescription of antibiotic therapy in primary care, was cited as a model for the introduction of this kind of tool.

There was no consensus within the group on the need to research and quantify the partner’s alcohol consumption in the event of a couple planning to become pregnant. The main point discussed by the experts in their comments was the role of the man during pregnancy follow-up consultations. According to the experts, women often consult alone. It is not possible to look for alcohol consumption by the partner. On the other hand, two experts felt that the doctor should initiate “*couple*” consultations, and disseminate information about the potential toxicity of alcohol consumption by the sire during embryogenesis.

The expert group reached a consensus on the need to investigate the use of various toxic substances on several occasions during pregnancy follow-up, and to record this in the patient’s medical record. Once again, the importance of training general practitioners was raised in the comments. However, some experts questioned the added time such an action would require during pregnancy follow-up consultations, which are often lengthy. The relevance of such an action was also questioned by one expert in the event of no follow-up being carried out.

Concerning the use of early detection and brief interventions on alcohol consumption among women and couples with a desire to become pregnant, using standardized questionnaires, there was no consensus within the expert group. In fact, several experts found the action too time-consuming. What is more, not all GPs are aware of, or trained in, this type of action.

**Figure 3:**
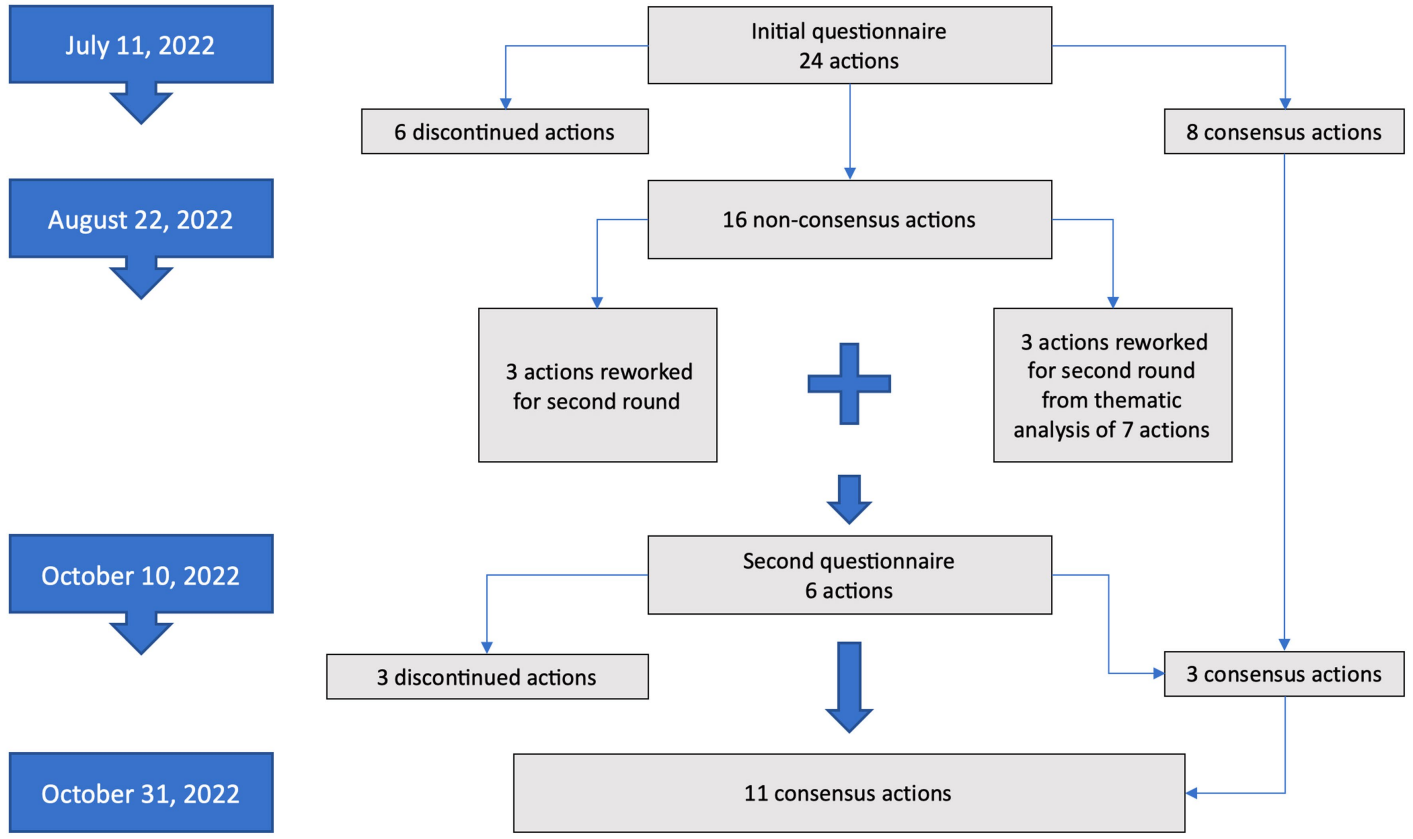
Summary of results.

There is a consensus that the minutes of the 4th-month prenatal interview with the midwife should be forwarded to the doctor monitoring the pregnancy, with the patient’s consent. For the experts, multidisciplinary follow-up multiplies the chances of identifying and monitoring women who consume alcohol during pregnancy (Figure 3).

## 4 Discussion

### 4.1 Strength and limits

Various studies on the prevention or identification of FASD using a Delphi round have been carried out in Australia (13) and South Africa (14). This study is original to France. The use of a Delphi round made it possible to convene a multidisciplinary group of experts, the majority of whom were general practitioners spread across the whole of Reunion Island. The selection of experts was the subject of numerous discussions between the researchers (AP-B, SL, and MS). This search for consensus is part of an action research project on the prevention and detection of fetal alcohol spectrum disorders in general practice. Regarding the high prevalence of FASD in general population, an agreement was found to select experts in primary care first. As with any selection process, there are limits to the participation of the people involved. The strong point of this method is the possibility of commenting anonymously, thus avoiding the effect of authority or opinion leaders (15). What is furthermore, the use of email to set up this study saved time and money.

Even if several professions working with FASD were represented on the expert panel, other players could have been involved, which constitutes a recruitment bias. Several reports emphasize the importance of identifying FASD in the course of legal proceedings. In 2018, an agreement was signed between the youth legal protection agency and the Reunion resource center to train its professionals in FASD (16). The absence of patients with FASD was also noted. Including other players in the expert group could have altered the results, potentially constituting a reproducibility bias.

### 4.2 Discussion of results

This research is part of an action research project. At present, four actions seem relevant for rapid implementation:

Consensus was based on two calculations: the median and the degree of convergence. After a thematic analysis, the experts’ free comments were also considered. These comments modulated the results of the calculations in the consensus decision.

The first action concerns the systematic introduction of a pre-conception consultation for women wishing to become pregnant. This non-compulsory but “strongly recommended” consultation is reimbursed at 70% (17). Its modalities have not been updated by the HAS since 2009, and were issued in response to a request from healthcare professionals following the abolition of the pre-nuptial consultation in 2007 (18). This consultation assesses medical, behavioral and social risks during pregnancy. It helps reduce the risks that can arise very early on during organogenesis, and later during embryonic and fetal development (19). A study carried out in 2015 on 392 patients suggested that only 15% had attended a pre-conception consultation, and that this constituted primiparous patients with a high socio-professional level (20).

The second action concerns the systematic identification of environmental factors during the child’s follow-up. Action levers for identifying FASD must take the environment into account. Studies of the psychosocial behavior of children with FASD have highlighted behavioral disorders that appear to be specific, resulting from an interaction between prenatal exposure to alcohol and environmental factors (21, 22). The impact of environmental factors has been demonstrated in the expression of other pathologies, notably psychiatric ones such as schizophrenia (23).

The third action proposed for implementation in the short term is the systematic distribution of an information document on the effects of fetal alcohol exposure when folic acid is prescribed pre-conceptionally or at the start of pregnancy. In 2016, in Nouvelle Aquitaine, a feasibility study was carried out on the distribution of this document by the general practitioner among women of childbearing age (24). The acceptability of the study and the information document was 100% with women, and this helped to encourage 34% of them to discuss their alcohol consumption with a healthcare professional. However, in this study, only 37% of women of childbearing age received the information leaflet. In our study, the experts suggested targeting the distribution of this type of document to women wishing to become pregnant or in early pregnancy.

Finally, the publication of an information leaflet for general practitioners on the identification of children with FASD, with contact details for associations, would also seem appropriate. The SAF France association, recognized by a number of learned societies, recommends that professionals be given the definition of FASD, as well as key figures such as incidence and patient association contacts, to help women in vulnerable situations. In 2021, a survey conducted by this association questioned 302 French general practitioners. Only 24% of them claimed to have precise knowledge of fetal alcohol spectrum disorders, and 1/3 of them said they did not systematically discuss alcohol consumption in early pregnancy (25). The publication of this brochure is a first step toward providing information and encouraging training.

## 5 Conclusion

This work led to a consensus on 11 actions to prevent and identify FASD in general practice. Four of these actions appear to be suitable for rapid implementation, following evaluation via feasibility studies. In the course of this work, several experts discussed the importance of including the pregnant woman’s partner in the prevention of FASD, but it is regrettable that no consensus was reached on an action to include him or her. The partner’s role is one of accompaniment and support during pregnancy. Moreover, the father’s alcohol consumption is also thought to increase the risk of FASD through epigenetic changes in sperm DNA (26). Finally, according to one study, 75% of FAS children have a biological father with an alcohol use disorder (27). Other research studies integrating the partner in the prevention of FASD would seem to be of interest.

## Data availability statement

The original contributions presented in the study are included in the article/Supplementary material, further inquiries can be directed to the corresponding author.

## Ethics statement

Ethical review and approval was not required for the study on human participants in accordance with the local legislation and institutional requirements. Written informed consent from the participants was not required to participate in this study in accordance with the national legislation and the institutional requirements.

## Author contributions

SL: Conceptualization, Investigation, Methodology, Project administration, Supervision, Validation, Visualization, Writing – original draft, Writing – review & editing. AP-B: Conceptualization, Data curation, Formal analysis, Investigation, Methodology, Visualization, Writing – original draft. BD: Writing – review & editing. TM: Writing – review & editing. FM: Writing – review & editing. CL: Writing – review & editing. CM: Writing – review & editing. MS: Conceptualization, Methodology, Project administration, Supervision, Validation, Writing – review & editing.

## Supplementary material

APPENDIX 1 SAF regional prevention action plan.

APPENDIX 1 Recruitment of experts.
