# Supplementary figures and images for: Actions to prevent and identify fetal alcohol spectrum disorders to be implemented in general practice: a consensus
Source: Front Med (Lausanne). 2024 Feb 5;11:1278973. doi: 10.3389/fmed.2024.1278973 (PMC10875990; doi:10.3389/fmed.2024.1278973)

1. Appendix 1: SAF regional prevention action plan


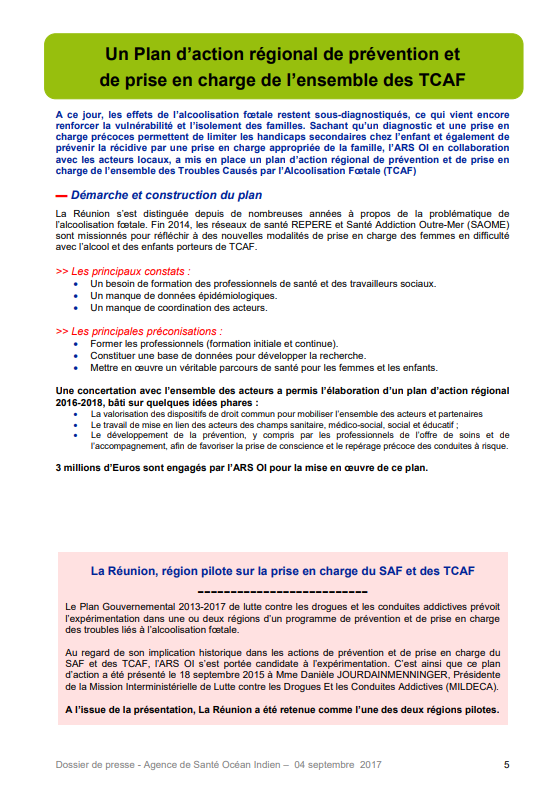


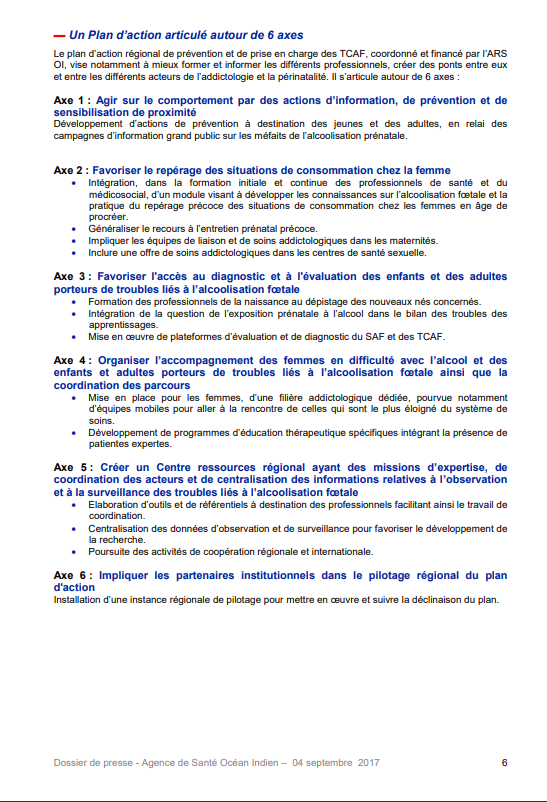

Supplement: APPENDIX 1 — SAF regional prevention action plan. [file Data_Sheet_1.docx]

1. Appendix 2: Recruitment of experts


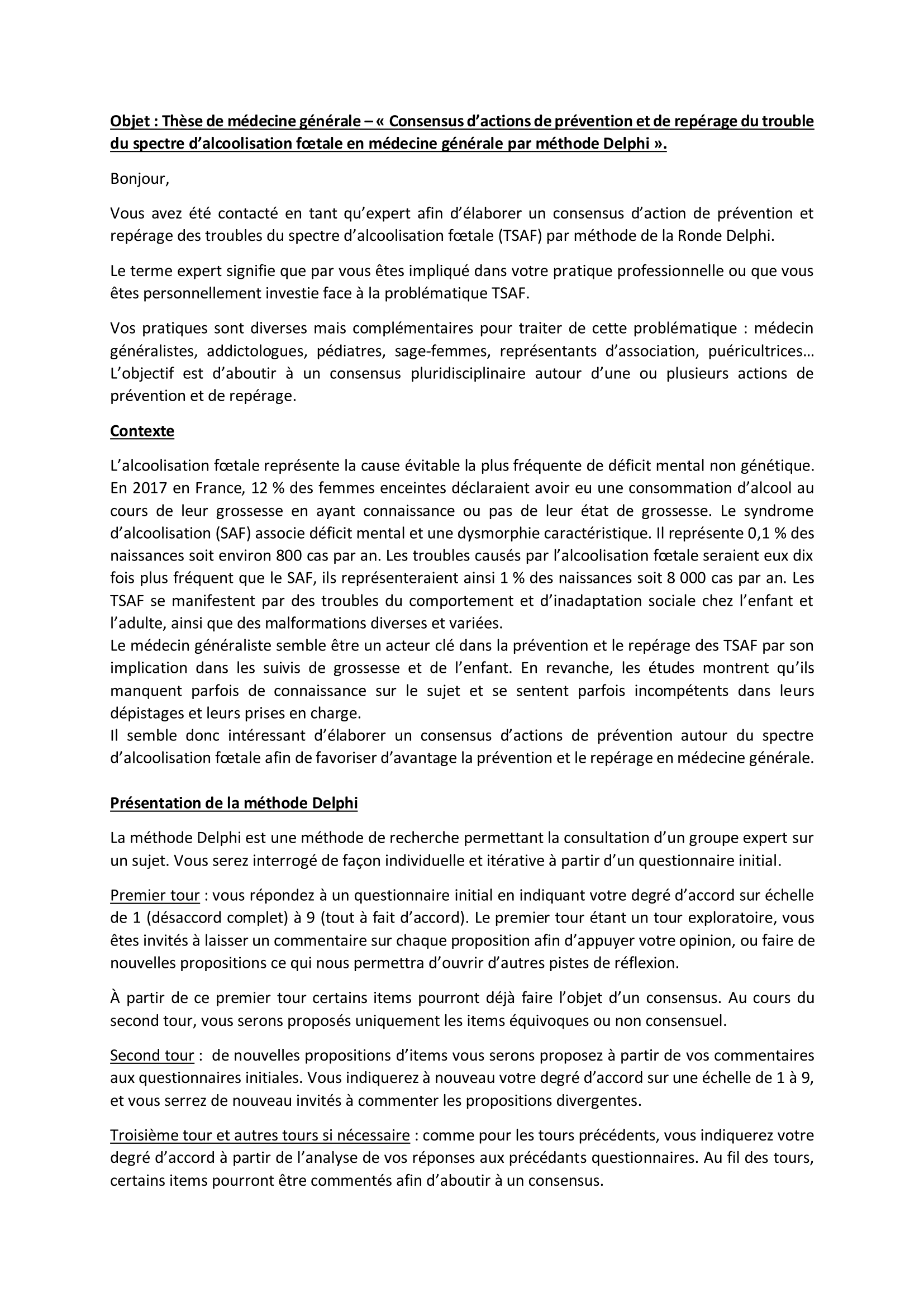


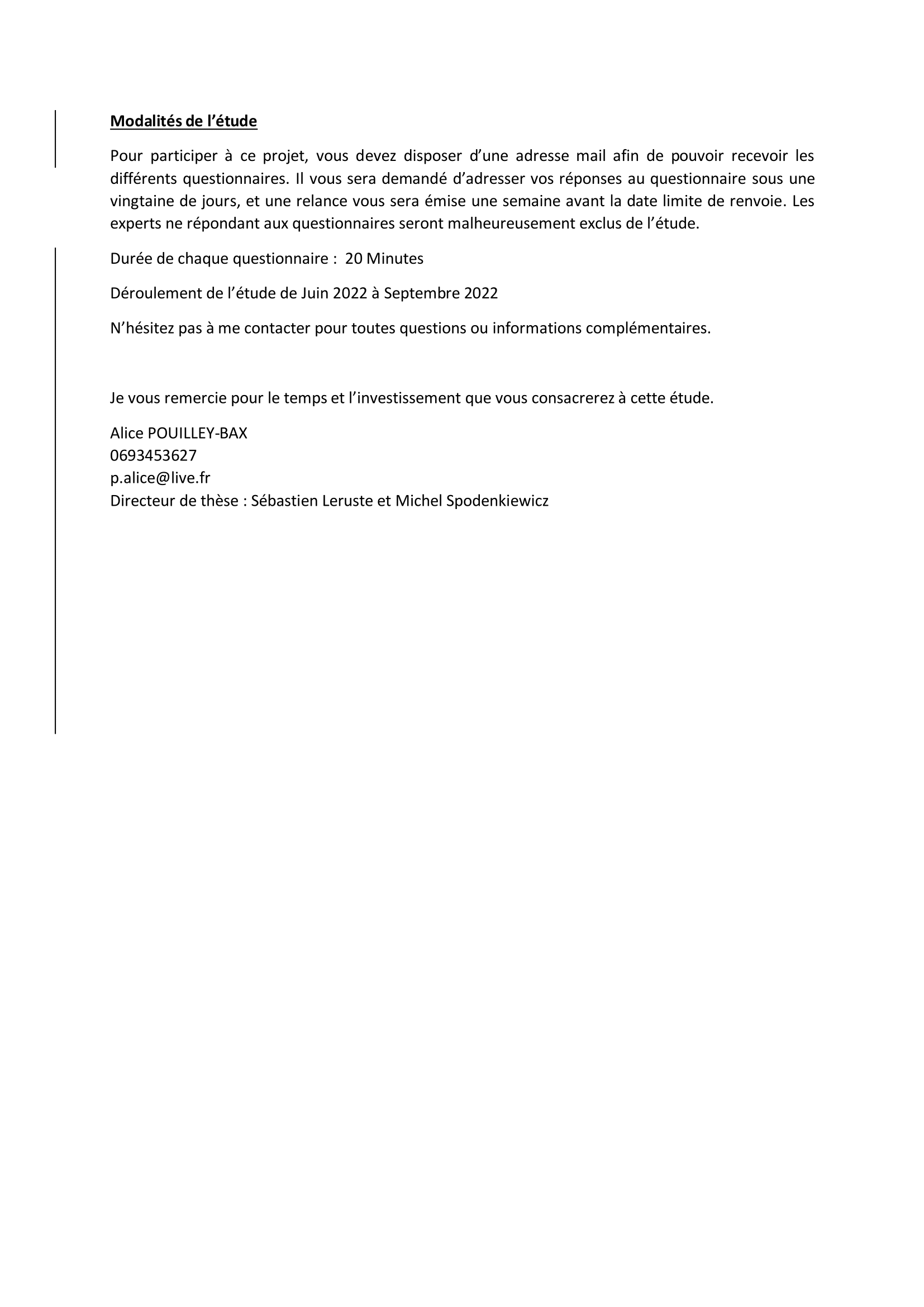

Supplement: APPENDIX 1 — Recruitment of experts. [file Data_Sheet_2.docx]
